# Museum genomics reveals the Xerces blue butterfly (*Glaucopsyche xerces*) was a distinct species driven to extinction

**DOI:** 10.1098/rsbl.2021.0123

**Published:** 2021-07-21

**Authors:** Felix Grewe, Marcus R. Kronforst, Naomi E. Pierce, Corrie S. Moreau

**Affiliations:** ^1^ Grainger Bioinformatics Center, Field Museum of Natural History, Chicago, IL 60605, USA; ^2^ Department of Ecology and Evolution, University of Chicago, Chicago, IL 60637, USA; ^3^ Department of Organismic and Evolutionary Biology, Harvard University, Cambridge, MA 02138, USA; ^4^ Department of Entomology, Cornell University, Ithaca, NY 14853, USA; ^5^ Department of Ecology and Evolutionary Biology, Cornell University, Ithaca, NY 14853, USA

**Keywords:** Lepidoptera, Lycaenidae, conservation, extinction, museomics, ancient DNA sequencing

## Abstract

The last Xerces blue butterfly was seen in the early 1940s, and its extinction is credited to human urban development. This butterfly has become a North American icon for insect conservation, but some have questioned whether it was truly a distinct species, or simply an isolated population of another living species. To address this question, we leveraged next-generation sequencing using a 93-year-old museum specimen. We applied a genome skimming strategy that aimed for the organellar genome and high-copy fractions of the nuclear genome by a shallow sequencing approach. From these data, we were able to recover over 200 million nucleotides, which assembled into several phylogenetically informative markers and the near-complete mitochondrial genome. From our phylogenetic analyses and haplotype network analysis we conclude that the Xerces blue butterfly was a distinct species driven to extinction.

## 1. Introduction

Understanding human impacts on biodiversity are essential for conservation. Determining how insect species and populations are being affected by pesticide use, land-use modification and climate change are all active areas of research (reviewed in [1]), but we still understand relatively little about how insects are affected overall [2]. One of the most iconic insect extinctions in the United States was the loss of the Xerces blue butterfly (*Glaucopsyche xerces*) from the costal sand dunes of San Francisco, California, USA in the early 1940s. Urban development and disturbance of sandy soils caused the local loss of several species of *Lupinus* and *Lotus*, particularly *Lo. scoparius* (Deerweed), its preferred larval host plant, and the resulting habitat change is thought to have brought about its extinction [3]. Its decline also coincided with the introduction of *Linepithema humile*, the Argentine ant, into the region, and it has been proposed that this invasive ant may have contributed to species loss by outcompeting native ant species that tend and protect the caterpillars of *G. xerces.* However, *L. humile* is known to tend lycaenid larvae in other cases, and several studies have suggested that they may function similarly to other ant symbionts of lycaenid larvae (e.g. [4,5]).

Despite only being formally described by Boisduval in 1852 [6] and declared extinct less than 100 years later in the 1940s, quite a lot is known about the biology of *G. xerces*. The species exhibited an unusual degree of variability in wing patterning, and Williams [7] and Downey & Lange [8] detailed morphological wing variation and genitalic structure, egg to adult development, larval parasites, major flight period and food plant preference. However, the question remained whether *G. xerces* was indeed a distinct species, subspecies or potentially just an isolated population of the widespread Silvery Blue, *Glaucopsyche lygdamus*, which has a range extending across the western United States and Canada. Downey & Lange [8, p. 165] note:

The genitalia of *G. xerces* are very similar to those of *G. lygdamus* (Dbldy.) On the basis of the male genitalia alone, *xerces* should be assigned subspecific status under *lygdamus*. However, there are differences between these species in larval stages, adult wing maculation, and ecology. In addition, occasional specimens of *G. lygdamus behrii* (Edw.) are taken in the areas where *xerces* occurred, and hybrids have never been detected. We are of the opinion that they are closely related but separate species.

Since the last specimens of *G. xerces* were seen alive in the 1940s, we turned to museum specimens to address this question. Although natural history museum collections are essential biodiversity repositories, preservation for molecular research was not a consideration in the past [9]. Museum genomics is really only now becoming viable owing to the short sequencing reads available from many next-generation sequencing technologies, allowing for the sequencing of highly fragmented DNA. We applied this approach to a museum specimen of the Xerces blue to assess whether it is, indeed, a genetically distinct lineage, in which case we would conclude that the Xerces blue is truly extinct.

## 2. Material and methods

A portion of a specimen of *G. xerces* collected in 1928 was used to extract DNA and the voucher is available in the scientific holdings of the Field Museum of Natural History. Single-end 150 bp sequencing libraries were prepared, adapters were ligated onto the DNA fragments and the library was sequenced on an Illumina MiSeq platform.

Raw sequences were processed, and four commonly used phylogenetic markers (nuclear genes 28S ribosomal RNA (*28S*), histone H3 (*H3*), and the mitochondrial *CO1-tRNA-CO2* (the cytochrome oxidase 1 marker region including the adjacent tRNA and parts of cytochrome oxidase 2)) were identified from the genomic sequence data, as well as the nearly complete mitochondrial genome. To infer phylogenies based on each successful marker region, we downloaded existing sequences from the BOLD and Genbank databases and all available complete mitogenome sequences of nine Lycaenidae from Genbank, in addition to two Riodinidae and two Nymphalidae as outgroups.

Phylogenetic inference was conducted using maximum likelihood and the GTR + G model of molecular evolution in RAxML v. 7.2.8 [10]. We conducted three sets of phylogenetic analyses: (i) *CO1* of *Glaucopsyche*, (ii) individual and combined analyses of *CO1*, *CO1-trnL-CO2*, *28S* and *H3* for Polyommatinae and (iii) full mitogenome analysis of nine Lycaenidae and outgroups (electronic supplementary material, table S1). A haplotype network was estimated using the *CO1* barcoding marker for all available sequences of *G. lygdamus* and *G. xerces*, and the sister to this clade, *G. lycormas*, to calculate a minimum spanning network. Full details of the methods, as well as additional analyses, are provided in the electronic supplementary material.

## 3. Results

**Figure 1.**
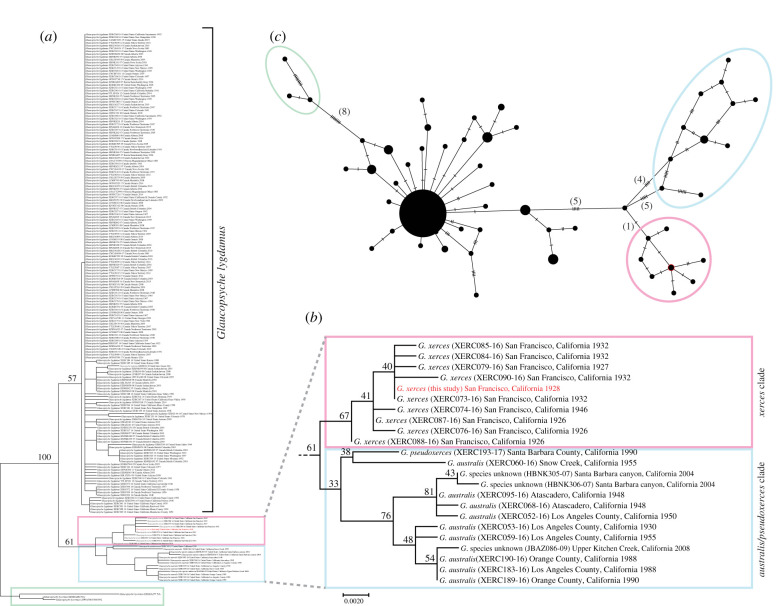
(*a*) Phylogenetic tree of *Glaucopsyche*. The tree was reconstructed by maximum likelihood inference of the *CO1* barcoding mitochondrial marker gene. Three *G. lycormas* sequences were used as an outgroup. *Glaucopsyche xerces* of this study is highlighted in red. Bootstrap values of main clades are indicated near each node. The detailed tree is presented in electronic supplementary material, figure S3. (*b*) Expansion of the *xerces* and *australis/pseudoxerces* clades of the phylogenetic tree in (*a*). Bootstrap support values are indicated near each node. Taxon labels indicate species names, BOLD Process IDs, collection locations and collection years. (*c*) Haplotype network of all *Glaucopsyche* calculated from the *CO1* barcoding marker used in the phylogeny of (*a*). Mutations are indicated by dashes and numbers in brackets. Ellipses highlight the clusters of *G. xerces* (pink), *G. australis/pseudoxer*ces (light blue) and *G. lycormas* (green). Red circle indicates the node of the network that included *G. xerces*.

The *G. xerces* DNA from the 93-year-old museum specimen was highly degraded, but we were able to recover 210 182 214 nucleotides of the Xerces blue butterfly genome (electronic supplementary material, table S2). The gene-based maximum likelihood phylogenetic trees all recover *G. xerces* as sister to a *G. australis/pseudoxerces* clade with modest bootstrap support and these are sister to the *G. lygdamus* clade (figure 1*a*,*b*; electronic supplementary material, figure S3). We also recovered *G. lycormas* as sister to *G. xerces* + *G. australis/pseudoxerces* and *G. lygdamus*.

The results of our haplotype network generated from the *CO1* barcode alignment (not including the three outgroup taxa) containing 197 *Glaucopsyche* sequences show higher mitogenomic divergence in the network separating *G. xerces* and *G. australis/pseudoxerces* from all other *G. lygdamus* (figure 1*c*). The average estimates of evolutionary distance based on the *CO1* barcoding gene also support these findings. Pairwise distances calculation of these sequences resulted in an average intraspecific mitogenomic divergence for *G. lygdamus* of 0.15% and for individuals of the *xerces* and *australis/pseudoxerces* clade 0.32 and 0.71%, respectively (electronic supplementary material, table S3). Both indicate that *G. xerces* (and *G. ausralis/pseudoxerces*) are distinct evolutionary clades.

**Figure 2.**
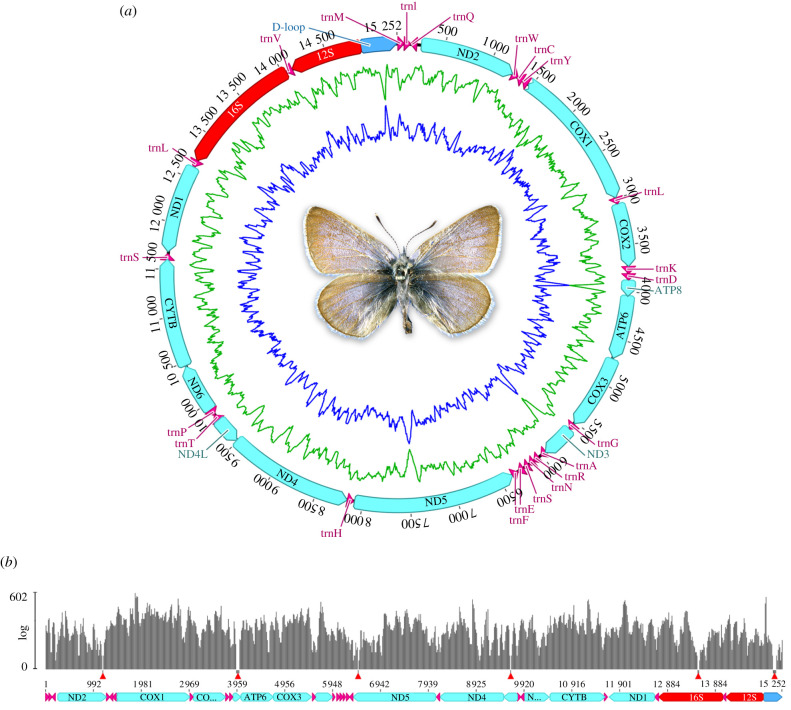
(*a*) Circular representation of the mitogenome of *G. xerces*. Protein-coding genes are highlighted in cyan, transfer RNA genes in magenta, ribosomal RNA genes in red, and the D-loop region in blue. Inner blue and green lines represent the CG and AT content, respectively. (*b*) Coverage histogram of the *G. xerces* mitogenome. Grey bars represent the genome coverage in a logarithmic scale. Red triangles point to six uncovered regions.

We assembled 15 252 bp of the *G. xerces* mitochondrial genome, with a GC content of 17.6% (figure 2*a*). We were able to assemble almost the entire mitogenome minus six small gaps in the *atp8*, *ND2*, *ND5*, *trnL*, *16S* rRNA genes and the D-Loop region (figure 2*b*). When comparing the *G. xerces* mitogenome to other lepidopteran mitogenomes we find the gene arrangements in continuous assemblies were identical (electronic supplementary material, figure S4). Although there were no other *Glaucopsyche* mitogenomes available on Genbank, we combined the protein-coding gene sequences from the *G. xerces* mitogenome with the gene sequences from all other available Lycaenidae mitogenomes to reconstruct a robust phylogenetic tree (electronic supplementary material, figure S5). In these analyses, we consistently recovered *G. xerces* grouping with other species of Polyommatinae within the Lycaenidae. Full details of the results are provided in the electronic supplementary material and sequence data have been deposited in Genbank (accession no. MW677564) and SRA (accession no. PRJNA705167).

## 4. Discussion

Museomics, or coupling museum specimens with genomic technologies, is permitting novel questions to be addressed that often cannot be answered in any other way. One example is sequencing specimens of extinct species, since fresh or living material is not an option [11]. In this study, we investigated whether the Xerces blue butterfly, *G. xerces*, was a distinct species or simply represented a slightly diverged population of the widespread Silvery Blue butterfly, *G. lygdamus*. This has implications for both conservation and potentially reintroduction. If *G. xerces* represented an isolated population of *G. lygdamus*, this would mean that humans did not cause the extinction of a distinct species, and that reintroduction could be sourced from one or more extant populations.

Using next-generation sequencing of a 93-year-old museum specimen of the Xerces blue butterfly and coupling this with additional sequences available on public databases, we showed that *G. xerces* was in fact a distinct species. This is in agreement with differences noted by Downey & Lange [8] that the larval stages, adult wing maculation, and ecology differ between these species. We also found the *G. xerces* clade as sister to a clade of *australis/pseudoxerces*. These two clades are sister to *G. lygdamus*.

Although the work reported here was not motivated by interest in the ‘resurrection’ of an extinct species, and we were unable to assemble the majority of the nuclear genome, our data provide the first step in determining the genetic differences between these species. In addition, de-extinction of the Xerces blue would require not only the recapitulation of the genetic diversity of the species, but also the reestablishment of the host plants it feeds on and potentially the reintroduction of protective symbiotic ant species to guard the larvae. Before de-extinction efforts are considered for this species, it will be important to consider the ecological and evolutionary costs and benefits [12]. The Xerces blue butterfly is an icon for insect extinction and conservation, and the question remains whether the financial and time investment in potentially resurrecting this species outweigh the investment in protecting other butterfly species and habitats that are currently in sharp decline, such as those of the El Segundo Blue, *Euphilotes battoides allyni* [13,14], or the Karner Blue, *Lycaeides melissa samuelis* [15,16].

## Data accessibility

Sequence data have been deposited in NCBI's Genbank (mitogenome accession no. MW677564) and SRA (BioProject ID: PRJNA705167).
